# Circulating microRNAs: a new era in early detection of oral squamous cell carcinoma (OSCC) — a systematic review and meta-analysis

**DOI:** 10.1186/s43046-026-00393-4

**Published:** 2026-08-10

**Authors:** Roshan Mustafa Pathan, Umadevi Subramanian, Jugraj Singh, Venkata Chandana Heshma Anumalasetty, Mukesh Ram Kumar Kommu, Vamsi Krishna Kondepati, Ponni Gayatri Twinkle Bade

**Affiliations:** 1 Siddhartha Medical College, Vijayawada, India; 2 Centre for Research Impact and Outcome (CRiO), Chitkara University, Rajpura, Punjab India; 3 Saveetha Dental College and Hospitals, Saveetha University, Chennai, India; 4 Verde Valley Medical Centre, Arizona, United States; 5 Dr. P.S.I Medical College, Gannavaram, India; 6 Rangaraya Medical College, Kākināda, India; 7 Government Medical College, Ongole, India; 8 Konaseema Institute of Medical Sciences and Research Foundation, Amalapuram, India

**Keywords:** Circulating microRNA (miRNA), Oral squamous cell carcinoma (OSCC), Liquid biopsy, Diagnostic biomarkers, Early cancer detection

## Abstract

**Background:**

The chances of oral squamous cell carcinoma (OSCC) being diagnosed at an early stage are very low, and this leads to a poor prognosis. The presence of circulating miRNAs (miRNAs) in blood or saliva is now a potential non-invasive diagnostic biomarker, although the diagnostic accuracy of the studies reported remains vastly different due to differences in biofluids, miRNA panels, disease spectrum, and platforms.

**Methods:**

We did a meta-analysis of diagnostic accuracy and a systematic review in accordance with PRISMA-DTA 2020 guidelines (PROSPERO: CRD420251274288). Articles that evaluated circulating miRNAs in any biofluid in diagnosing OSCC were taken into consideration. A Bayesian bivariate random-effects model was used to pool together the sensitivity, specificity, likelihood ratios, diagnostic odds ratios and the summary receiver operating characteristic (sROC) curves. Heterogeneity sources were analyzed using pre-constructed subgroup analysis and meta-regression.

**Results:**

The number of studies that were evaluated in totality was seventeen and seven were incorporated in the meta-analysis. The pooled *sensitivity* of the circulating miRNAs towards the detection of OSCC was *0.89 (95% CI: 0.77–0.95)* and the pooled *specificity* was *0.88 (95% CI: 0.75–0.95)*. It had a pooled positive likelihood ratio *(PLR)* of *7.25 (95% CI: 3.42–16.08)*, a negative likelihood ratio *(NLR)* of *0.13(95% CI: 0.05–0.27)*, and a diagnostic odds ratio *(DOR)* of *59.69(95%CI:14.33-209.95)*. Good performance in discriminating was also noted in the sROC curve with an area under the curve value of approximately 0.90. However, substantial between-study heterogeneity was observed (τ ≈ 0.93 for sensitivity; τ ≈ 0.83 for specificity), and the 95% credible intervals around the DOR were wide (14.33–209.95). Individual studies of multi-miRNA panels reported AUCs up to 0.98, though pooled subgroup estimates were limited by small study numbers and wide uncertainty.

**Conclusions:**

Circulating miRNAs and in particular multi-miRNA saliva-based profiles provide promising but heterogenous diagnostic accuracy for OSCC. While individual panel studies have reported high AUCs, the current pooled evidence—derived from only seven small, mostly case-control studies of advanced-stage disease—does not yet establish utility for true early detection or population screening.

## Introduction

Oral squamous cell carcinoma (OSCC) is the most common head and neck cancer and occurs due to stratified squamous epithelium of the mouth cavity and is among the most common causes of morbidity and mortality of cancers anywhere in the globe [1]. A significant percentage of patients are already diagnosed with the disease at stage III or IV when the treatment becomes more complicated, toxicity is more significant, and the five-year survival rate decreases significantly [2]. Early diagnosis contributes greatly to good prognosis yet the current methods of diagnosis are not effective in the detection of early or silent disease.

The progressed or frank lesions can be identified through routine oral examination (visual examination and palpation) but in most instances cannot detect the disease at an early stage or submucosally [2]. Incisional biopsy and further histopathological examination is the diagnostic gold standard in case the suspicious lesion has been detected. Despite its extreme specificity, biopsy is invasive, related to patient discomfort and risks of the procedure, and can be unable to sample the most dysplastic area [3]. It is important to note that screening with biopsy at the population level cannot be carried out on or repeated among people who have oral potentially malignant disorders (OPMDs) [4]. These deficiencies justify the application of a diagnostic tool that is non-invasive and repeatable and can be applied in the detection of the condition at its initial phases and in subsequent follow-ups.

Liquid biopsy, i.e. use of tumor-derived molecular material released in easily accessible body fluids, is the promising approach that has emerged in cancer detection. The microRNAs (miRNAs) are one of the analytes of interest [5]. MiRNAs are non-coding RNA molecules that are short and control gene expression in post-transcriptional pathways and are key participants in the oncogenic events, such as proliferation, apoptosis, invasion, and metastasis [6]. Misregulation of particular miRNAs in OSCC is an early tumorigenic event. After the release into circulation miRNAs are extraordinarily stable in saliva and blood because of their encapsulation in extracellular vesicles or because of their binding to protective proteins [7]. Changes in miRNA expression patterns of patients with OSCC have also been demonstrated and can be utilized as early diagnostic biomarkers [8].

The findings are promising; however, the diagnostic performance reported by the studies is extremely varied, with some studies having modest to near-perfect sensitivity and specificity [9]. This heterogeneity may be attributed to differences in biofluid selection (saliva vs. blood), selection of biomarkers (single miRNAs vs. multi-miRNA panels) and the selection of control groups (healthy vs. benign or pre-malignant lesions) and the platform of analysis (quantitative PCR, microarray or sequencing-based analyses) [10]. This results in a disagreement with regard to the optimal miRNA signature, sampling technique or real diagnosis capacity of circulating miRNAs in OSCC.

In order to overcome these doubts, we performed a meta-analysis of a systemic review and diagnostic test accuracy to introduce the available evidence of circulating miRNAs in OSCC. By synthesizing the existing data, we hoped to assess the performance of diagnostic systems in general, the most effective biomarkers and biofluids, the locations of heterogeneity and whether the current evidence can be used to develop a simple, low-cost and repeatable saliva-based or blood-based assay to early predict and better manage OSCC.

## Methodology

### Study design and registration

We used the PRISMA-DTA 2020 checklist of reviews that combine diagnostic-test-accuracy information. We put the entire plan in PROSPERO before we started any work under ID CRD420251274288. The plan gave the precise question, the people to be included, the outcomes that were important and how we would analyse the numbers, which were predetermined to make the review transparent and prevent selective reporting.

### Eligibility criteria (PICO framework)

We included the rules in the PICO list but we limited it to the oral squamous cell carcinoma (OSCC).

#### Population (P)

We incorporated studies whereby those whose OSCC diagnosis was performed with the microscope have been recruited. Acceptable control groups were healthy volunteers, patients with oral potentially malignant disorders or patients with oral lichen planus. We have filtered out those that combined head-and-neck cancers and did not give results separately of OSCC.

#### Index test (I)

We searched on circulating microRNAs in saliva, serum, plasma or any other body fluid. The tests were required to employ molecular tests such as quantitative real time PCR or droplet digital PCR.

#### Comparator (C)

In whatever case the original study, the comparison group had to be healthy controls or patients with non malignant oral disease (OPMD or OLP), whichever the original study utilized.

#### Outcomes (O)

We extracted sensitivity, specificity, positive likelihood ratio, negative likelihood ratio, diagnostic odds ratio as well as the area under the ROC curve.

We included diagnostic accuracy studies, case - control studies, cohort studies and cross-sectional studies that provided sufficient numbers to construct a 2 × 2 table or at least one of the above measures of accuracy and Only studies reporting diagnostic accuracy of circulating miRNAs in saliva, serum, or plasma were eligible for quantitative pooling. Tissue-based studies were included for qualitative context only.

#### Rationale for including multiple study designs

Though the key research question was on the diagnostic accuracy of circulating miRNAs in the diagnosis of OSCC, the available literature has two different types of study design: (1) diagnostic accuracy studies, which report paired sensitivity and specificity rates or give adequate information to generate a 2 × 2 table, and (2) observational studies, which measures the expression of miRNAs in cases and controls but do not report diagnostic test accuracy rates. The different types of design answer a complementary aspect of the overall question. Diagnostic accuracy studies directly measure the accuracy with which a miRNA-based test accurately diagnoses or rules out OSCC - this is the essential evidence required to be clinically validated. Although not intended to assess diagnostic performance per se, observational expression studies provide important contextual data about the biological behaviour of circulating miRNAs, such as their direction and magnitude of dysregulation, their relationship with clinicopathological features (e.g., stage, grade, lymph node involvement) and their mechanism of action in oral carcinogenesis. By combining the two designs thus, a more thorough synthesis is possible than with either of the two: quantitative pooling answers the question of does it work? and narrative integration answers the question of why does it work? To analyze them, the two types of studies were processed in different streams: the quantitative meta-analysis (primary analysis) incorporated studies on diagnostic accuracy, and the qualitative synthesis (secondary analysis) incorporated only observational studies. None of the observational studies added some data to the pooled sensitivity, specificity, or other accuracy measures.

### Search strategy

A systematic literature search was conducted across PubMed/MEDLINE, Embase, and Google Scholar. The search was last performed on December 1 2025.

The PubMed/MEDLINE search was constructed using a combination of Medical Subject Headings (MeSH) terms and free-text terms across three conceptual blocks:

1. circulating microRNA-related terms,
2. oral squamous cell carcinoma-related terms, and
3. diagnostic accuracy-related terms.

The three search strings used were:

#### Search string 1

(“MicroRNAs”[Mesh] OR “microRNA*”[Title/Abstract] OR “miRNA*”[Title/Abstract] OR “miR”[Title/Abstract] OR “circulating miRNA”[Title/Abstract] OR “cell-free miRNA”[Title/Abstract] OR “exosomal miRNA”[Title/Abstract] OR “serum miRNA”[Title/Abstract] OR “plasma miRNA”[Title/Abstract])

AND

(“Mouth Neoplasms”[Mesh] OR “oral squamous cell carcinoma”[Title/Abstract] OR “OSCC”[Title/Abstract] OR “oral cancer”[Title/Abstract] OR “squamous cell carcinoma of the mouth”[Title/Abstract] OR “oral cavity cancer”[Title/Abstract] OR “tongue squamous cell carcinoma”[Title/Abstract])

AND

(“Early Detection of Cancer”[Mesh] OR “Screening”[Mesh] OR “early detection”[Title/Abstract] OR “early diagnosis”[Title/Abstract] OR “screening”[Title/Abstract] OR “diagnostic accuracy”[Title/Abstract] OR “sensitivity”[Title/Abstract] OR “specificity”[Title/Abstract] OR “ROC”[Title/Abstract] OR “liquid biopsy”[Title/Abstract] OR “non-invasive diagnosis”[Title/Abstract])

#### Search string 2

((((“circulating microRNA*”[Title/Abstract] OR “circulating miRNA”[Title/Abstract] OR “plasma miRNA”[Title/Abstract] OR “serum miRNA”[Title/Abstract] OR “exosomal miRNA”[Title/Abstract])

AND (“oral squamous cell carcinoma”[Title/Abstract] OR “OSCC”[Title/Abstract]))

AND (“early detection”[Title/Abstract] OR “early diagnosis”[Title/Abstract] OR “screening”[Title/Abstract] OR “diagnostic”[Title/Abstract])))

#### Search string 3

(“MicroRNAs”[Mesh] OR “microRNA*”[Title/Abstract] OR “miRNA*”[Title/Abstract] OR “miR”[Title/Abstract] OR “circulating miRNA”[Title/Abstract] OR “cell-free miRNA”[Title/Abstract] OR “exosomal miRNA”[Title/Abstract] OR “serum miRNA”[Title/Abstract] OR “plasma miRNA”[Title/Abstract])

AND

(“Mouth Neoplasms”[Mesh] OR “oral squamous cell carcinoma”[Title/Abstract] OR “OSCC”[Title/Abstract] OR “oral cancer”[Title/Abstract] OR “squamous cell carcinoma of the mouth”[Title/Abstract] OR “oral cavity cancer”[Title/Abstract] OR “tongue squamous cell carcinoma”[Title/Abstract])

AND

(“Early Detection of Cancer”[Mesh] OR “Screening”[Mesh] OR “Prognosis”[Mesh] OR “early detection”[Title/Abstract] OR “early diagnosis”[Title/Abstract] OR “screening”[Title/Abstract] OR “diagnostic accuracy”[Title/Abstract] OR “sensitivity”[Title/Abstract] OR “specificity”[Title/Abstract] OR “ROC”[Title/Abstract] OR “liquid biopsy”[Title/Abstract] OR “non-invasive diagnosis”[Title/Abstract] OR “prognosis”[Title/Abstract] OR “prognostic value”[Title/Abstract])

The major Embase search strategy included

(‘microRNA’/exp OR ‘circulating microRNA’/exp OR ‘cell-free microRNA’/exp OR ‘exosome’/exp OR ‘serum microRNA’/exp OR ‘plasma microRNA’/exp OR microRNA*:ti, ab OR miRNA*:ti, ab OR miR: ti, ab) AND (‘mouth tumor’/exp OR ‘oral squamous cell carcinoma’:ti, ab OR OSCC: ti, ab OR ‘oral cancer’:ti, ab OR ‘tongue squamous cell carcinoma’:ti, ab OR ‘oral cavity cancer’:ti, ab) AND (‘cancer screening’/exp OR ‘early cancer detection’/exp OR ‘early diagnosis’/exp OR ‘diagnostic accuracy’/exp OR ‘sensitivity and specificity’/exp OR ‘receiver operating characteristic’/exp OR ‘liquid biopsy’/exp OR ‘cancer prognosis’/exp OR early detection: ti, ab OR screening: ti, ab OR diagnostic: ti, ab OR ‘ROC curve’:ti, ab OR ‘non-invasive diagnosis’:ti, ab OR prognosis: ti, ab OR ‘prognostic value’:ti, ab).

Records retrieved: The search yielded 1955 records from PubMed/MEDLINE and 782 records from Embase. After removal of duplicates, 1091 records were available for screening.

### Study selection process

All the retrieved records were contained in a reference management system and duplicates were eliminated. Titles and abstracts were screened by two reviewers who screened them independently to exclude ineligible studies. Articles were evaluated based on set inclusion and exclusion criteria. Any disagreements were discussed or consulted with a third reviewer. To record the study selection process, a PRISMA flow diagram explaining the reasons why the full text was excluded was used.

### Data extraction

All screening, data extraction, and risk-of-bias assessments were performed independently by two reviewers, with disagreements resolved by consensus or arbitration by a third reviewer. The information that was extracted contained author information, publication year, country, study design, population characteristics, sample size, miRNA(s) that were evaluated, biofluid type, platform used to detect the miRNA, reference standards, patterns of expression and also diagnostic accuracy measures. Where possible, incomplete data were obtained by contacting corresponding authors.

### Risk of bias and quality assessment

The reviewing process was done by two reviewers independently and any discrepancies settled by consensus or a third reviewer. Considering the dual-stream nature of this review, study appraisal was conducted with two validated appraisal instruments depending on the type of study. The QUADAS-2 (Quality Assessment of Diagnostic Accuracy Studies-2) tool was used to assess diagnostic accuracy studies by evaluating the risk of bias, the tool involves four domains, namely patient selection, index test, reference standard, and flow and timing. The Newcastle-Ottawa Scale (NOS) was used to evaluate observational non-diagnostic studies by appraising three areas: the selection of participants, group comparability, and outcome/exposure evaluation.

The quality assessment score alone was not used to eliminate studies as there were few eligible studies. Rather, descriptively, risk-of-bias assessments were applied to put the entire strength of the evidence base into context. None of the studies included was considered as having a critical risk of bias.

The primary meta-analysis could not use stratified analyses that would be based on risk-of-bias ratings because of the few studies of diagnostic accuracy (*n* = 7). Nevertheless, risk-of-bias evaluations were considered with study estimates in forest plots to determine whether the low-quality studies had a disproportionate effect on the pooled findings. They recognize this limitation and risk-of-bias-based sensitivity analyses should be used as an analytical step in future meta-analyses that have a larger sample of studies on diagnostic accuracy.

### Outcomes definition

The primary outcomes were pooled sensitivity and specificity of circulating miRNAs for OSCC diagnosis. Secondary outcomes included PLR, NLR, DOR besides AUC derived from summary receiver operating characteristic (sROC) curves. Subgroup analyses were predefined based on biofluid type (saliva vs. blood), biomarker format (single miRNA vs. multi-miRNA panels), disease spectrum (OSCC vs. healthy controls - OSCC vs. OPMD/OLP) or detection platform (qRT-PCR vs. ddPCR). Only studies evaluating circulating miRNAs in saliva, serum, or plasma were eligible for quantitative pooling; tissue and FFPE-based studies were retained for qualitative biological context but excluded from the diagnostic accuracy meta-analysis.

### Statistical analysis

R version 2026.01.0 + 392 (R Foundation for Statistical Computing, Vienna, Austria) was used to perform all the analyses in the Rstudio environment.

#### Bivariate random-effects model — rationale

The main analytic model chosen was a bivariate random-effects model since it simultaneously models both sensitivity and specificity and maintains the negative relationship between them intrinsic to cross-studies. The bivariate model contrasts univariate methods (which combine sensitivity and specificity separately) with between-study heterogeneity in both parameters, taking into consideration the precision of the individual study estimates, and avoiding the undue impact of small studies with extreme values. Logit-transformed sensitivity and specificity were used with random effects to adjust to the expected differences in diagnostic thresholds, assay platforms and population characteristics among studies.

#### Primary meta-analysis

The primary pooled estimates of sensitivity and specificity were found by using a Bayesian bivariate random-effects model. The bayesmeta package with JAGS called through the rjags package was used to perform this analysis. All the model parameters were specified with weakly informative priors: the logit-transformed mean sensitivity and specificity were given normal priors N(0, 10 2), and the between-study standard deviations were given vague uniform priors. The Gelman-Rubin diagnostic (R-hat < 1.05) was used to measure model convergence. Two chains were executed and at least 20,000 iterations and a burn-in of 10,000 iterations per chain. The summary estimates are presented in the form of posterior medians with 95% credible intervals (CrIs).

#### Secondary analyses

The sensitivity and specificity pooling of frequentist bivariate random-effects meta-analysis were conducted on the mada package, whereas positive likelihood ratio (PLR), negative likelihood ratio (NLR), and diagnostic odds ratio (DOR) were estimated with the metafor package. The bivariate model parameters were used to construct the summary receiver operating characteristic (sROC) curves and the area under the sROC curve (AUC) was obtained to summarize the overall diagnostic performance. The sROC curve graphically illustrates the trade-off between sensitivity and specificity when comparing the studies and gives a threshold-independent measure of test accuracy.

#### Heterogeneity assessment

Between-study heterogeneity was measured by the I 2 statistic (calculated based on Cochran Q-statistic) and the between-study variance (tau). Predefined subgroup analyses were to investigate the expected heterogeneity (40%-80) based on biofluid (saliva vs. blood), biomarker format (single miRNA vs. multi-miRNA panels), disease spectrum (OSCC vs. healthy controls vs. OPMD/OLP), and detection platform (qRT-PCR vs. ddPCR). The metafor package was used to do meta-regression in order to estimate the impact of these covariates on sensitivity and specificity.

#### Publication bias

The funnel plot asymmetry test by Deek was used to evaluate publication bias with a significant level at *p* < 0.10. This was pre-specified because the statistical power of this test with a small number of included studies (*n* = 7) is known to be reduced.

## Results

### Study selection

**Fig. 1:**
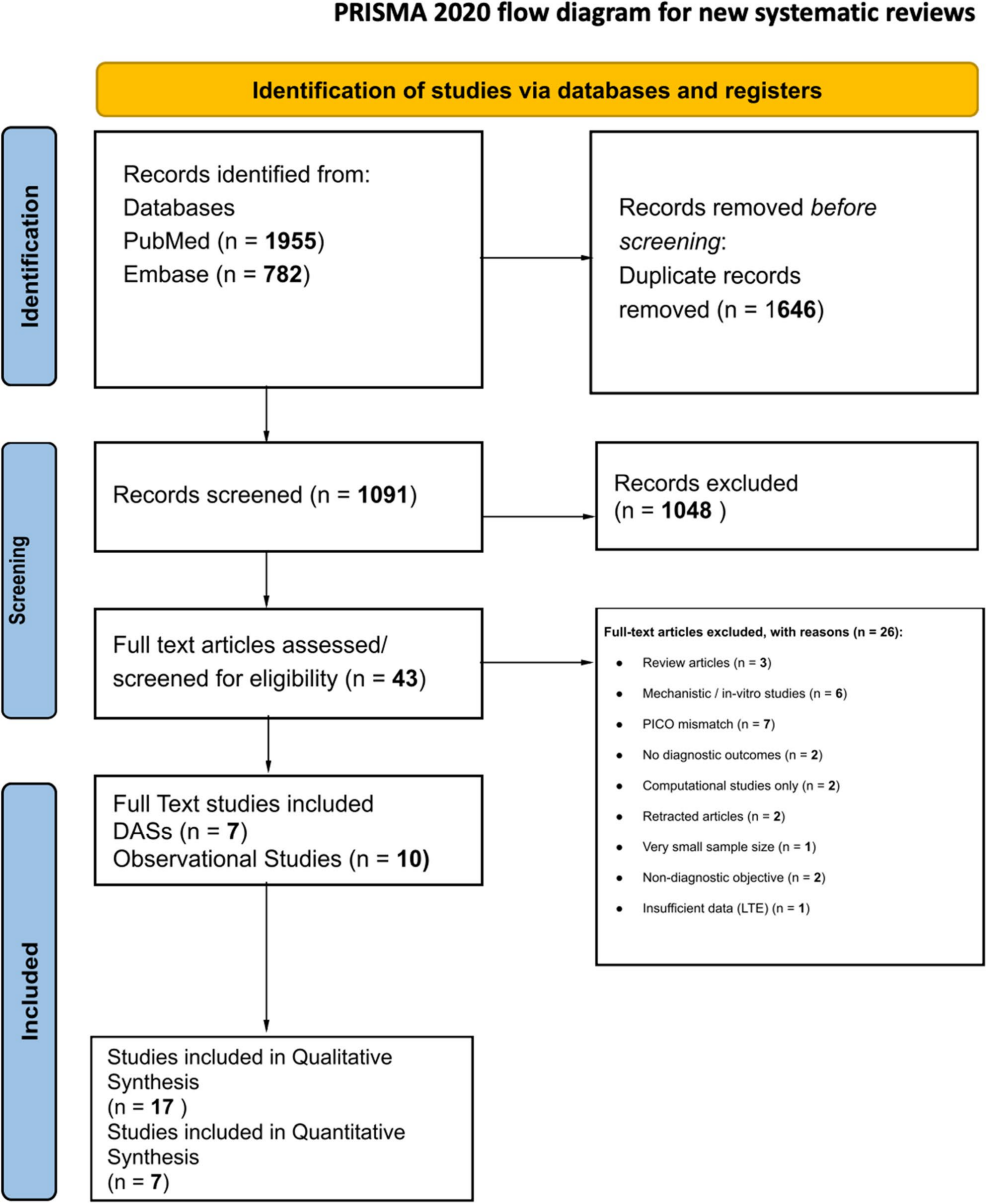
PRISMA-DTA 2020 flow diagram of study selection

We adhered to PRISMA 2020 guidelines and depicted the steps in Fig. 1. In the databases we identified 2737 references - no additional trials were found in registries. We read titles and abstracts of 1091 references after we deleted 1646 duplicates. We discounted 1048 since they did not respond to our question. We then read 43 full papers. We eliminated 26 of them due to obvious reasons as indicated in the PRISMA flow chart. The number of articles that met all the requirements yet were included in the qualitative review was 17 − 7 of those articles contained the data needed to conduct the meta analysis as well.

### Study characteristics

A total of 17 studies were reviewed in full − 7 of them examined the sensitivity of micro-RNA markers in the detection of oral cancer - the remaining 10 merely reported the level of micro-RNA in patients. No randomised trials were found, which is typical of a review that focuses on biomarkers, as opposed to drugs or surgeries. The articles were written between 2018 and 2025 and were of China, India, Iran, Italy, Germany, Australia in addition to Egypt which provided the review with a good geographic coverage.

Study sample sizes ranged from 16 to 396 participants. Among the 17 included studies, 13 evaluated circulating miRNAs in saliva, serum, or plasma, while 4 evaluated tissue-based samples (3 fresh tissue and 1 FFPE). Tissue-based studies were retained for qualitative biological context but were excluded from the quantitative diagnostic accuracy meta-analysis. Of the circulating miRNA studies, most enrolled adult patients with OSCC; two studies additionally included patients with OPMD or OLP, and one large multicenter study enrolled patients with head and neck cancer (HNC) broadly. Because of heterogeneous reporting of multi-group designs (e.g., studies reporting OSCC, OPMD, OLP, and healthy controls separately), aggregate case and control totals were not tabulated.

Of the 17 included studies, 6 evaluated saliva (5 saliva-only, plus 1 study that compared plasma and saliva), 5 evaluated serum, 3 evaluated plasma, and 4 evaluated tissue-based samples (3 fresh tissue and 1 FFPE). Tissue and FFPE studies were retained for qualitative biological context but were excluded from the quantitative diagnostic accuracy meta-analysis because they do not represent circulating miRNAs.

In all groups of diagnostic miRNA assays, miR-191-5p had the most stable level - its values changed almost insignificantly between cancer and control samples. miR-484 was the second in terms of stability. The measurements remain more stable when miR-191-5p is used alone or in combination with miR-484 as a normalizer as opposed to using older controls such as U6 along with improved accuracy and repeatability of the diagnosis.

In cancers of the mouth, the microRNA called miR-1290 is always found at high levels (up regulated). It assists the tumour to grow since it suppresses the genes that would normally keep the tumour at bay. Concurrently, the cell is deprived of a set of protective microRNAs - miR-223, miR-99a, miR-133a, miR-375, miR-200b and miR-580. The decline in their level (down regulated) eliminates the natural inhibition on growth, enhances resistance to treatment and an aggressive nature to the tumour. Tests to measure oral cancer microRNAs in the blood have an average sensitivity of approximately 85–90% and specificity of approximately 75–85% in clinical studies. Multiple microRNAs combined on panels are more effective than tests depending on one marker. The microRNAs with the abnormal levels prevent or enhance messenger RNAs that promote tumor progression such as STAT3, IGF1R and mTOR pathway associated genes, thus proving their usefulness as diagnostics.

### Risk of bias assessment

As the study was a mixture of diagnostic accuracy studies and observational studies, we addressed the risk of bias in each group separately by using another, validated checklist that complies with its design as per Cochrane in addition to PRISMA-DTA recommendations.

#### Diagnostic accuracy studies 

We used QUADAS-2 form, which examines four items - how patients were selected, how the new test was administered, how the reference test was administered and how patients flowed through the study. Most items rated low risk had many studies. Patient selection was the only area of common concern with some authors adopting a case - control design or selecting patients in a non consecutive manner. Very few studies had high ratings of patient selection or flow plus timing, nearly always due to insufficient information in the report or small sample size. The index test and the reference standard were almost always considered to be low risk, since the tests were done properly or OSCC was proven through histopathology. Those ratings are indicated in the QUADAS-2 traffic light plot and the bar charts.

#### Observational studies: 

We applied the Newcastle - Ottawa Scale that addresses three questions - the selection of participants, the comparability of groups but also the outcome or exposure. Most of the studies were low to moderate risk. Outcome assessment and selection were high throughout the set. The comparability item was more diverse, as the various studies did not correct the potential confounders. None of the observational studies was excluded due to critical bias - it was considered to be sufficient to perform qualitative synthesis and to compare expressions of interest. Because of the small number of diagnostic accuracy studies, a formal sensitivity analysis stratified by risk of bias was not feasible. Qualitatively, visual inspection of the forest plots did not suggest that studies with higher risk of bias (e.g., non-consecutive patient selection) produced substantially different sensitivity or specificity estimates than lower-risk studies, though this observation is limited by low power.

All of these ratings indicate that the evidence is, overall, sound, and also indicate areas where methods varied across studies.

### Qualitative synthesis (narrative results)

Diagnostic accuracy research and observational studies all concur that microRNAs both in blood and tissue were significantly altered. in oral squamous cell carcinoma. Our pooled numbers were only concerned with the effectiveness of those markers in identifying disease - but numerous studies unable to be combined were providing valuable clinical and biological information.

Mapping work, which mapped expression, revealed that the change in miRNA levels is in the expected pattern of cancer-driving molecules and cancer-blocking molecules. Tumour-blocking miRNAs, such as miR-let-7a and miR-99a, were detected in smaller amounts in carcinoma than in normal tissue - in several studies their levels were still lower in lesions that were premalignant compared to those that were malignant indicating they might play an early role in transformation. The miRNAs - miR-21, miR-1290, miR-155, miR-191 and miR-494 were repeatedly found to be elevated in serum, saliva, plasma and tissue implicating them in growth, invasion and spreading.

In addition to mere separation of patients and controls, low miR-let-7a and miR-99a levels were associated with higher stage, lack of differentiation, further lymph node metastases and reduced survival, which cannot be expressed in a pooled diagnostic statistic. Also by tissue assays miR-21 abundance was found to vary in one oral site versus another, indicating variability within or between tumours.

**Fig. 2:**
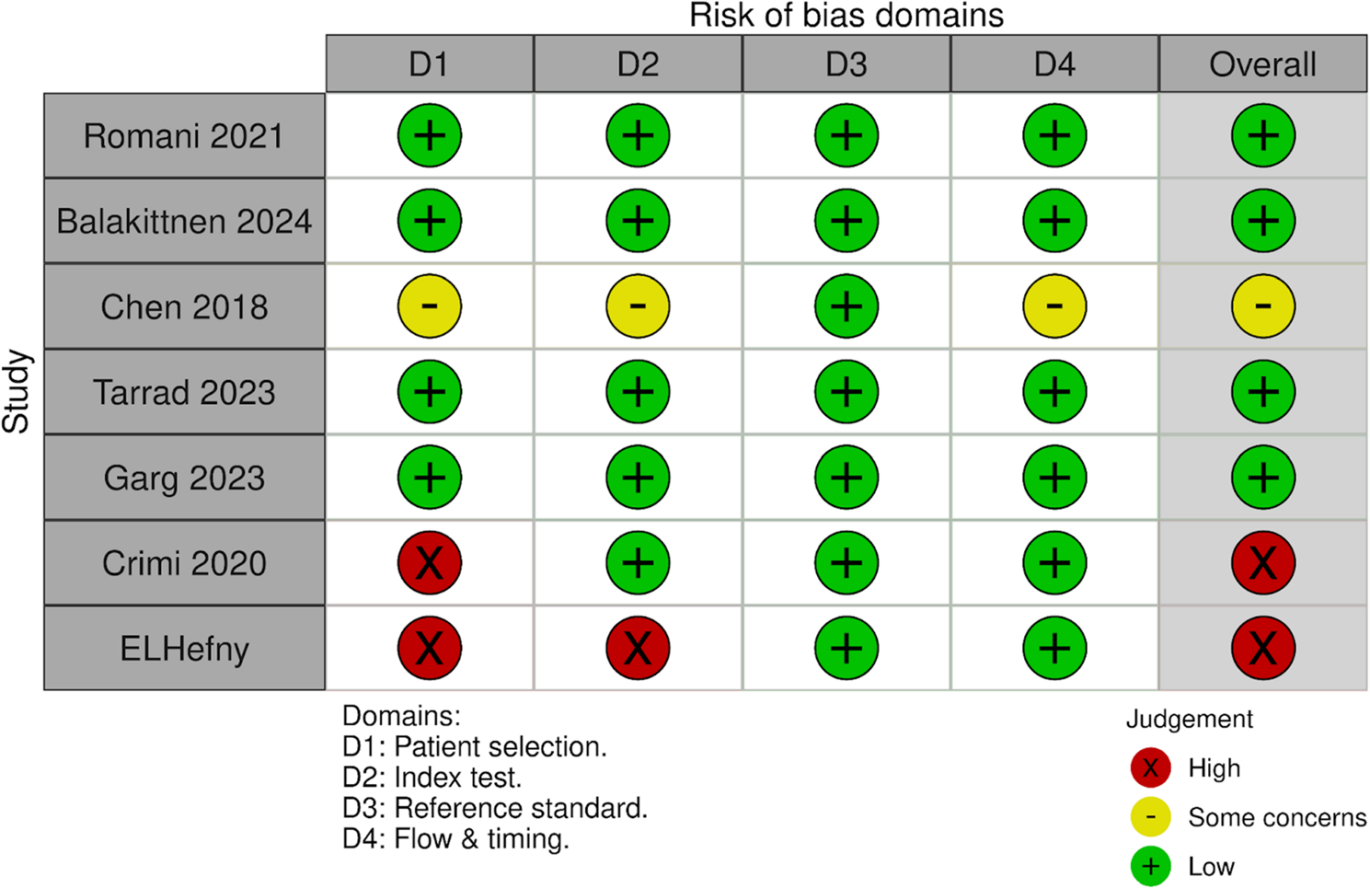
risk of bias and applicability assessment across included studies

Disagreements between reports are primarily due to biology, rather than study design. Research involving potentially malignant diseases or inflammatory lesions such as oral lichen planus tended to give lower specificity, as some of the molecular signature is also present in carcinoma. The exploratory and mechanistic papers also gave functions to these miRNAs in radiation response, apoptosis regulation and cell-cycle timing - these end points cannot be included in a meta analysis but are essential to biological knowledge (Fig. 2).

Combined, the qualitative data support the aggregated diagnostic findings - that the miRNA alterations observed in OSCC are biologically sensible, have clinical implications and are dependent on context and thus should be considered as early molecular markers on the path to oral cancer.

### Quantitative synthesis (meta-analysis results)

**Fig. 3:**
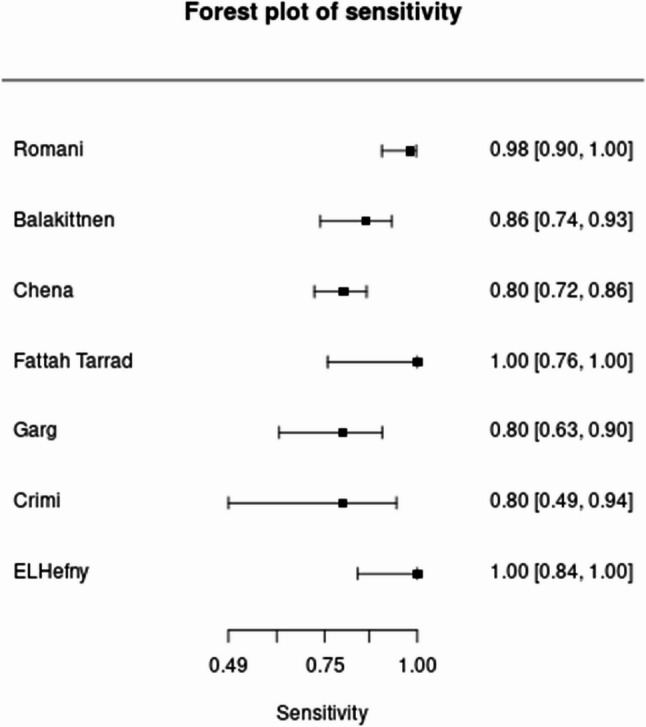
Forest plot of sensitivity for circulating miRNAs in the detection of oral squamous cell carcinoma

**Fig. 4:**
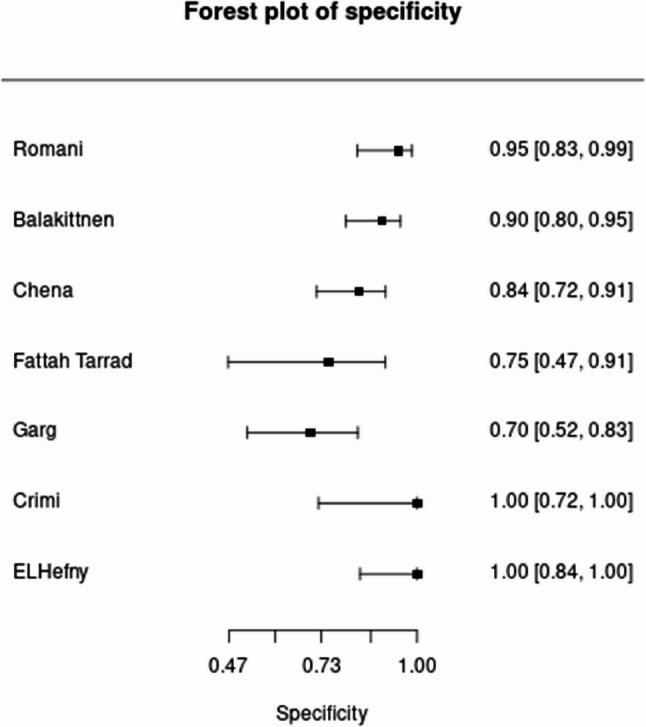
Forest plot of specificity for circulating miRNAs in the detection of oral squamous cell carcinoma

**Fig. 5:**
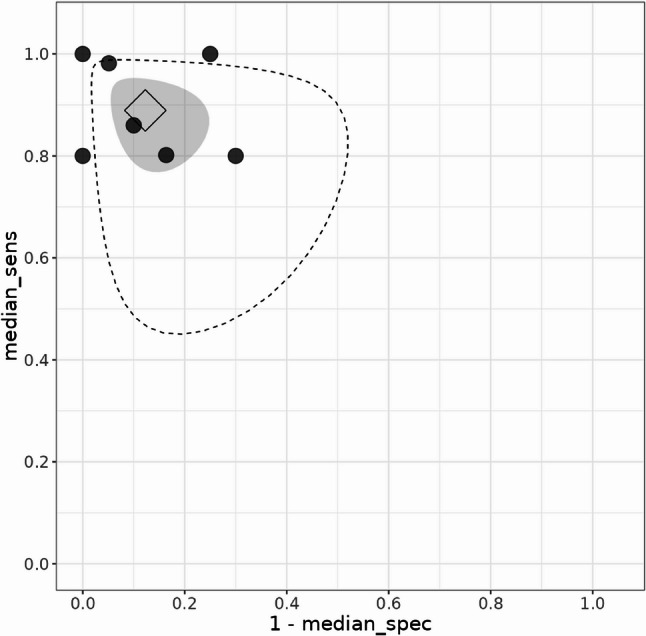
Summary receiver operating characteristic (sROC) curve for circulating miRNAs in the detection of oral squamous cell carcinoma

We pooled the accuracy of the tests using the microRNAs of different samples in all the eligible studies attempting to identify oral squamous cell carcinoma or head and neck squamous cell carcinoma. The numbers (Figs. 3, 4 and 5) were plotted using a Bayesian bivariate random effects model to display the pooling - sensitivity and specificity forest plots and the sROC curve. The summary sensitivity reached 0.89 (95% CI 0.77–0.95) the summary specificity reached 0.88 (95% CI 0.75–0.95). The pooled positive likelihood ratio (PLR) equalled 7.25 (95% CI 3.42–16.08). The pooled negative likelihood ratio (NLR) equalled 0.13 (95% CI 0.05–0.27). The pooled diagnostic odds ratio equalled 59.69 (95% CI 14.33–209.95). Obvious heterogeneity within the studies emerged - the between-study standard deviation of logit transformed sensitivity was 0.93 plus and the between-study standard deviation of logit transformed specificity was 0.83. Such values are the basis of the random effects approach. Comprehensively, the findings indicate that the circulating microRNAs clearly separate patients with and without OSCC or HNSCC.

### Meta-regression of included studies

**Fig. 6:**
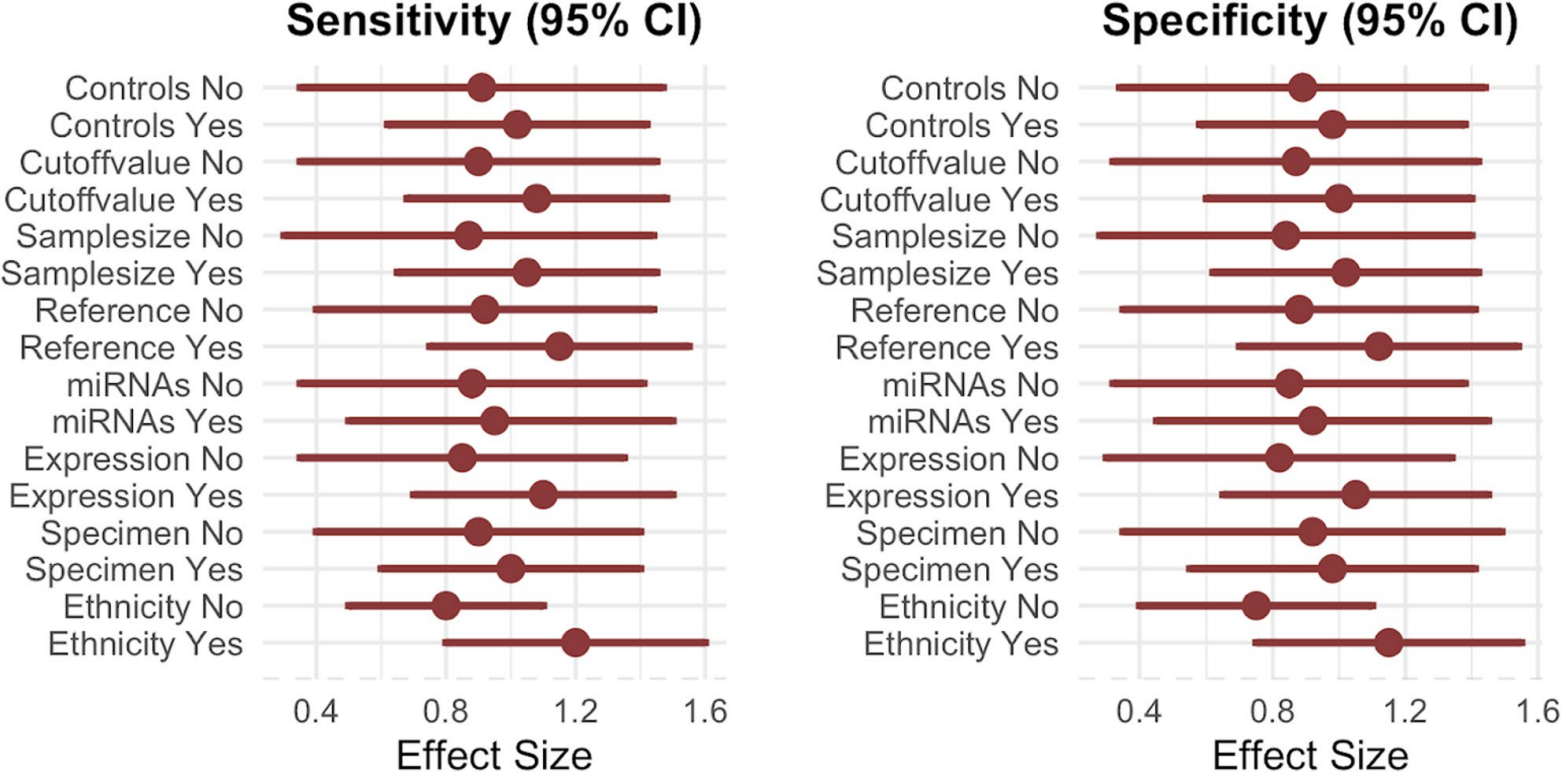
Univariable random-effects meta-regression of study-level covariates affecting diagnostic performance. Covariates examined included biomarker format (single miRNA vs. multi-miRNA panel), panel size (number of miRNAs per panel), and biofluid type (saliva, serum, or plasma). Each dot represents an individual study estimate on the log diagnostic odds ratio [log(DOR)] scale, weighted by study precision; horizontal lines indicate 95% confidence intervals

Meta-regression was performed to evaluate whether the use of multi-miRNA panels influenced overall diagnostic performance compared with single-miRNA assays. Univariable random-effects meta-regression using log(DOR) showed that panel type was the strongest moderator of diagnostic performance. Multi-miRNA panels showed a positive trend toward higher diagnostic odds ratios than single-miRNA assays (β = 1.694, SE = 0.932, z = 1.817, *p* = 0.069; 95% CI -0.133 to 3.521), explaining 60.3% of between-study heterogeneity (R² = 60.3%), with residual heterogeneity reduced to τ² = 0.573 and I² = 48.9%. By contrast, panel size (β = 0.113, *p* = 0.632) and biofluid type (QM = 1.254, *p* = 0.534) did not significantly explain heterogeneity (Fig. 6; Table 1).

**Table 1:** Characteristics of the studies included in this analysis

Author, Year	Country / Study Design	Population & Sample Size	miRNA / Biomarker	Sample Source	Cancer Type	Expression in Cases	Expression in Controls	Key Findings
Romani et al., 2021 [11]	Italy / Multi-stage discovery–validation	94 (55 OSCC, 39 healthy)	miR-106b-5p, miR-423-5p, miR-193b-3p	Saliva supernatant	OSCC	miR-423-5p: mean 2⁻ΔCq = 0.349	miR-423-5p: mean 2⁻ΔCq = 0.138	3-miRNA panel achieved AUC 0.98; sensitivity 97.4%, specificity 94.2%; miR-423-5p predicted poor DFS
Balakittnen et al., 2024 [12]	Australia / Two-phase study	110 (50 °C, 60 healthy)	8-miRNA signature	Saliva	OC / OPMD	ΔCt-based upregulation	Lower ΔCt expression	AUC 0.954; sensitivity 86%, specificity 90%
Chen et al., 2018 [13]	China / Case–control	176 (121 OSCC, 55 healthy)	miR-99a	Serum	OSCC	Downregulated	Higher expression	AUC 0.911; sensitivity 80.2%, specificity 83.6%
Tarrad et al., 2023 [14]	Egypt / Prospective diagnostic	24 (12 OSCC, 12 controls)	LINC00657, miR-106a	Saliva	OSCC / OLP	LINC00657 FC 8.66 ± 7.18	FC 1.09 ± 0.12	Sensitivity 100%, specificity 75%
Garg et al., 2023 [15]	India / Case–control	60 (30 OSCC, 30 controls)	miR-21, miR-184	Saliva	OSCC / OPMD	miR-21 FC 3.7 ± 2.0	FC 1.0	miR-21 AUC 0.858
Crimi et al., 2020 [16]	Italy / Pilot validation	20 (10 OSCC, 10 healthy)	miR-375-3p, miR-133a-3p	Plasma	OSCC	Significantly reduced	Higher expression	miR-375-3p AUC 0.96; specificity 100%
El Hefny et al., 2024 [17]	Egypt / Case–control	20 OSCC, 20 OLP, 20 healthy	miR-93, miR-412-3p	Saliva	OSCC	Upregulated	Low baseline	AUC 0.958–1.000
Garajei et al., 2023 [18]	Iran / Retrospective	40 (20 OSCC, 20 healthy)	miR-21-5p, miR-429	Tissue	OSCC	Median 1.09	Median 0.57	miR-21-5p AUC 0.867
Priya et al., 2021 [19]	India / Tissue study	40 samples	miR-21	FFPE tissue	OSCC	Lower Ct (higher expression)	Higher Ct	Overexpression in 73% OSCC
Pastorino et al., 2022 [20]	Multicenter / INHANCE	396 cases, 396 controls	miR-151-3p	Plasma	HNC	ΔCt 6.89	ΔCt 7.68	Early-stage diagnostic utility
Seyedhoseini et al., 2025 [21]	Iran / Cross-sectional	112 samples	miR-let-7a	Serum	OSCC / OLP	1.55 ± 1.19	7.02 ± 4.10	Downregulation linked to stage
Bagheri Shirvan et al., 2024 [22]	Iran / Clinical study	32 participants	miR-1290	Serum	OSCC	4.44 ± 3.73	1.34 ± 1.99	Significant upregulation
Emami et al., 2020 [23]	Iran / Case–control	100 participants	miR-155, miR-191, miR-494	Serum	OSCC	1.08–2.4-fold ↑	Baseline	OncomiRs; miR-191 highest fold change
Hofmann et al., 2022 [24]	Germany / Exploratory	16 participants	Exosomal miRNA panels	Plasma, saliva	HNSCC	Altered profiles	Normal profiles	Exploratory discrimination
Jia et al., 2022 [25]	China / Retrospective	100 samples	miR-580	Tissue	OSCC	Downregulated	—	Radiosensitivity via STAT3
Zheng et al., 2015 [26]	China / Observational	84 cases	miR-491-3p	Tissue	Tongue SCC	Downregulated (73.8%)	—	Poor prognosis marker
Nagdeve et al., 2025 [27]	USA / Technical study	Laboratory samples	miR-31	Serum (spiked)	Model system	—	—	—

*OSCC* oral squamous cell carcinoma, *OC* oral cancer, *OPMD* oral potentially malignant disorders, *OLP* oral lichen planus, *HNC* head and neck cancer, *SCC* squamous cell carcinoma, *FC* fold change, *Ct* cycle threshold, Δ*Ct* delta cycle threshold, *DFS* disease-free survival, *AUC* area under the receiver operating characteristic curve

Expression values are reported as described in the original studies and include relative expression, fold change, ΔCt values, or Ct values, as applicable. Lower Ct or ΔCt values indicate higher miRNA expression. Diagnostic performance metrics (AUC, sensitivity, specificity) are derived from receiver operating characteristic (ROC) analyses

Studies using tissue samples or laboratory-based model systems are included for biological context but were not pooled in the diagnostic accuracy meta-analysis. Exploratory and technical validation studies were analyzed qualitatively only

### Subgroup analyses

Subgroup analyses showed that saliva tests give better diagnostic results than blood tests, especially when multiple miRNAs are measured together (Table 2). Panels that combine multiple markers always detected disease and ruled out healthy people more reliably than single miRNAs (Table 3). Most accurate was oral squamous cell carcinoma vs. healthy controls - accuracy decreased but still provided useful clinical data, when carcinoma was compared to oral potentially malignant diseases or lichen planus which reflected actual biological disparities (Table 4). Droplet digital PCR is much more accurate and makes better diagnoses but it is expensive and in limited supply, preventing it from becoming part of routine practice - most studies have remained with qRT-PCR to be used in everyday clinical and research practice.

**Table 2:** Biofluid type (saliva vs. blood)

Measure	Saliva	Blood
Sensitivity	0.89 (95% CI: 0.69–0.97)	0.79 (95% CI: 0.54–0.91)
Specificity	0.84 (95% CI: 0.63–0.93)	0.86 (95% CI: 0.58–0.96)
PLR	5.46 (95% CI: 2.25–12.53)	5.44 (95% CI: 1.71–21.98)
NLR	0.13 (95% CI: 0.04–0.39)	0.26 (95% CI: 0.11–0.59)
DOR	44.60 (95% CI: 7.44–196.03)	21.87 (95% CI: 3.60–142.08)
AUC (sROC)	≈ 0.90	≈ 0.87
Meta-regression	**Reference (higher accuracy)**	Contributed to heterogeneity

**Table 3:** Single miRNA vs. multi-miRNA panels

Measure	Single miRNA	Multi-miRNA Panel (2 to 8)
Sensitivity	0.79 (95% CI: 0.63–0.89)	0.92 (95% CI: 0.67–0.98)
Specificity	0.81 (95% CI: 0.57–0.93)	0.88 (95% CI: 0.65–0.95)
PLR	4.05 (95% CI: 1.79–10.93)	7.50 (95% CI: 2.48–17.67)
NLR	0.26 (95% CI: 0.14–0.53)	0.09 (95% CI: 0.03–0.39)
DOR	15.97 (95% CI: 3.81–56.96)	83.92 (95% CI: 10.54–407.87)
AUC (sROC)	~ 0.86–0.88	~ 0.94–0.96
Meta-regression	Reference category	**Significant contributor to heterogeneity** **(p < 0.05)**

Multi-miRNA panels ranged from 2 to 8 miRNAs across included studies

**Table 4:** OSCC vs. healthy controls only ⟂ OSCC vs. OPMD / OLP (± healthy controls)

Measure	OSCC vs. Healthy Controls only	OSCC vs. OPMD / OLP (± Healthy)
Sensitivity	0.86 (95% CI: 0.56–0.95)	0.85 (95% CI: 0.61–0.94)
Specificity	0.89 (95% CI: 0.65–0.96)	0.79 (95% CI: 0.54–0.91)
PLR	7.34 (95% CI: 2.17–20.51)	4.07 (95% CI: 1.72–9.07)
NLR	0.17 (95% CI: 0.05–0.53)	0.19 (95% CI: 0.07–0.55)
DOR	45.65 (95% CI: 5.56–243.09)	21.74 (95% CI: 3.81–88.74)
AUC (sROC)	≈ 0.93	≈ 0.89
Meta-regression	Reference category	**Disease spectrum significantly contributed to heterogeneity** **(p < 0.05)**

### Publication bias

**Fig. 7:**
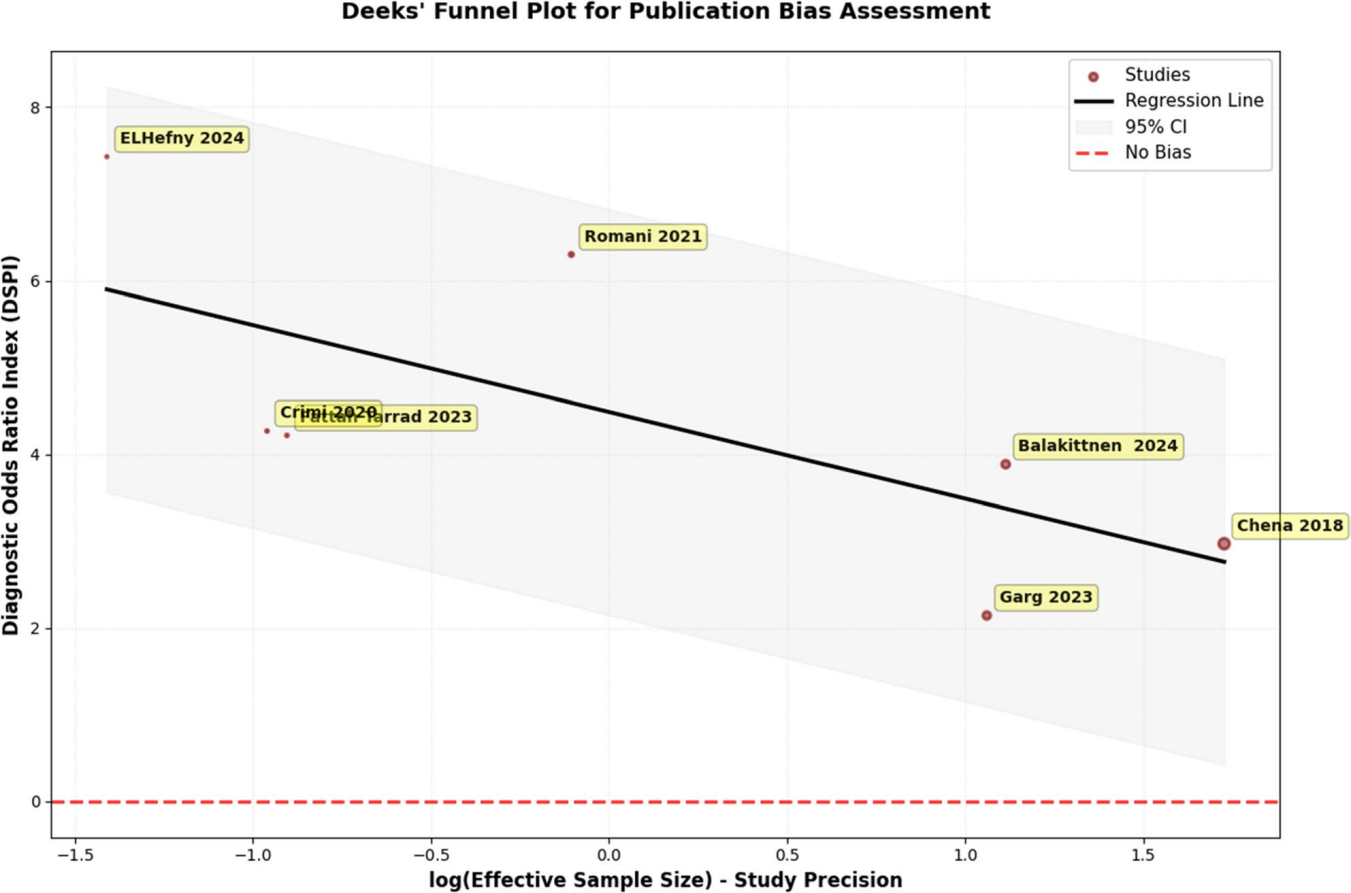
Assessment of publication bias using deeks’ funnel plot asymmetry test

Publication Bias Assessment (Fig. 7) - No significant imbalance was detected (*p* = 0.0864) - the miRNA biomarker meta analysis does not seem to be affected by publication bias. The regression slope (-1.0002) indicates that at each level of accuracy, studies are equally distributed on each side of the null line. Smaller trials (low ESS) have an upward bias to greater values in DSPI but the difference is not significant (t = -2.13). The evidence presented in the plot is well distributed and has an acceptable accuracy. Chen (2018, *n* = 171) provided the most extensive sample of patients other than ELHefny (2024) with a perfect sensitivity and specificity. Collectively, the review generates an evidence base that is very much objective, and there is no indication that the reports about diagnostic accuracy were selectively withheld.

## Discussion

Circulating miRNAs demonstrate strong diagnostic accuracy for oral squamous cell carcinoma, achieving a pooled sensitivity of 0.89 (95% CI: 0.77–0.95), specificity of 0.88 (95% CI: 0.75–0.95), PLR of 7.25, NLR of 0.13, and DOR of 59.69 [17]. The sROC polled AUC was 0.90. Nevertheless, there was a significant between-study heterogeneity (τ = 0.93 sensitivity, 0.83 specificity) and pooled estimates should be interpreted with caution. There is no standardised protocol on miRNA isolation into body fluids, and presence of oncogenic and tumour-suppressor miRNAs in pooled analyses also complicates interpretation [17, 28]. Two step approach, sensitive non-invasive screening and a specific confirmatory test are suggested as a strategy to ensure maximum clinical utility [17]. 

In line with previous narrative reviews [29], multi-miRNA panels have always performed better in comparison with single-marker tests in all bio fluids and assays platforms [11–16]. Individual studies of multi-miRNA panels reported AUC values ranging between 0.95 and 0.98 - significantly higher than conventional serum tumour marker CEA and CA19-9, which in head and neck cancer would typically have polled AUCs of 0.60–0.70 [11, 15, 19]. The results of individual panel performances were impressive: serum miR-99a produced AUC 0.911 with the sensitivity of 80.2 and specificity 83.6; salivary 8-miRNA signature in an individual study has achived an AUC 0.954 (sensitivity 86.0, specificity 90.0); and a 3-miRNA salivary panel had AUC in another study achieved an AUC of 0.98.

Across individual studies, saliva-based assays tended to achieve higher sensitivity (range 80–100%) than blood-based assays (approximately 80%), whereas blood-based assays showed superior specificity (range 84–100%) [11, 12, 14–16]. This anatomical gradient suggests direct tumour-derived miRNA shedding into saliva, whereas blood measurements may be subject to dilution and renal clearance. Similarly, when individual studies compared OSCC with healthy controls, reported sensitivities and specificities ranged from 80% to 98%; against OPMD or oral lichen planus comparators, sensitivity fell to between 70% and 90% [11–16]. This overlap likely reflects a shared molecular signature between premalignant and malignant lesions rather than assay imprecision; as such, these patterns could be clinically useful for stratifying patients with OPMD at highest risk of malignant transformation [12, 14, 15].

A coherent pattern of miRNA dysregulation across the studies included supported the biological validity of these results. Oncogenic miRNAs were persistently upregulated -miR-21 (2.7- to 3.7-fold), miR-1290 (increased in both tissue and serum), miR-151-3p (early-stage plasma), miR-155 (1.08-fold), and miR-191 (up to 2.4 Tumour-suppressor miRNAs, on the other hand, were suppressed: miR-let-7a, miR-99a (reducing with disease progression), miR-133a-3p (80–90% loss of plasma signal), miR-375-3p (100% specificity in single panel) [12, 16]. The most frequently reported oncogenic miRNA was miR-21 [11, 15, 19]. Concordant dysregulation was further validated in the studies that directly compared tissue and circulatory compartments, in which miR-21-5p was higher in both, which validated the usefulness of blood-based testing as a surrogate of tissue testing [30].

Platform comparisons qRT-PCR (used in most studies) was found to be comparable in the majority of studies in terms of performance (consistency 80–100% sensitive; 70–95% specific), although there is heterogeneity in methodological approaches to reference genes and normalisation strategies [16]. One study reported ddPCR as 80% sensitive and 100% specific with absolute quantification that was independent of calibrators [16]. Nevertheless, cross platform comparison could not be made solid because of the single ddPCR validation study. Previous systematic reviews of blood-based biomarkers to predict head and neck cancer have focused on protein-based markers or miRNAs in tissues - the present review is the first to combine diagnostic accuracy rates of circulating miRNAs in OSCC [31].

Circulating miRNAs exceed the technical performance criteria needed to be clinically utilized, especially in high-risk groups. Collection on saliva is painless, needs no professional equipment, and can be used to screen at home or at the population level, individual studies reported diagnostic performances ranging from AUC 0.85 to 0.98, with sensitivities of 80–100% and specificities of 75–95% as good or better than other established methods such as brush biopsy and narrow-band imaging. Nevertheless, standardisation of miRNA extraction and normalisation procedures are a significant obstacle to clinical translation and the cost-effectiveness data compared to a standard procedure are required to inform health policy, especially in resource-limited contexts [17, 32].

The review has strengths such as prospective registration, dual-stream synthesis that combines both diagnostic accuracy and observational evidence, predefined subgroup analysis by biofluid, biomarker format, disease spectrum, and detection platform, geographic diversity of six countries. The main limitations are the use of only cross-sectional and retrospective case-control designs (not allowing the consideration of performance in prospective screening environments), the presence of only advanced-stage cases (58% stage III-IV), small median sample sizes (approximately 60–100 participants), and insufficient attention to controlling confounding factors such as smoking, alcohol, and betel quid use. Most research based diagnostic cut-offs on internal ROC analysis, as opposed to external validation, which risks overfitting. Little information on premalignant lesions and no longitudinal studies followed up the OPMD patients with regard to malignant transformation were found [13, 16, 17]. The observed overlap in miRNA signatures between OSCC and OPMD/OLP—while reducing specificity in a diagnostic context—may be clinically useful for risk stratification. Patients with OPMD exhibiting an OSCC-like miRNA profile could represent a high-risk subgroup for malignant transformation, though longitudinal studies are needed to validate this.

Future research should be based on large-scale prospective cohort design with standardised miRNA panels, standard extraction methods, and externally verified diagnostic cut-offs. To fill in critical evidence gaps and to facilitate clinical translation, longitudinal monitoring of OPMD cohorts with circulating miRNA signatures, the evaluation of miRNA dynamics during therapy and follow-up, and larger studies in ddPCR validation are needed.

## Conclusion

This review and meta-analysis suggests that salivary and blood-based miRNA are strong, non-invasive way of diagnosing oral squamous cell carcinoma. Tests using those miRNAs were very accurate to diagnose in all the studies, especially when a group of miRNAs was measured, and when saliva was tested. The two combined data are sensitive and specific - the test can distinguish between persons with OSCC and healthy persons with a high degree of reliability - but the test can also distinguish between patients and individuals with precancerous lesions although the test is not so good at it. Where studies were sub-classified, the differences in accuracy were not due to the inability of the study design to emphasise the fact that these are markers of the earliest stages of tumour change but equally in the business of risk grading. qRT-PCR is the most practically feasible method of detection to date, but initial findings indicate that droplet digital PCR can give even more accurate information. Collectively, the findings suggest that a conglomerate of salivary miRNAs may emerge as a convenient method of screening and follow-ups of OSCC at an early stage. To validate the ideal panel and to transfer the marker to practice large prospective studies with the same protocol across centres would now be required.

## Data Availability

All data analyzed in this study were derived from published articles. Extracted datasets and analytical code are available from the corresponding author upon reasonable request.
